# Improving linear accelerator service response with a real‐time electronic event reporting system

**DOI:** 10.1120/jacmp.v15i5.4807

**Published:** 2014-09-08

**Authors:** Jeremy D. P. Hoisak, Todd Pawlicki, Gwe‐Ya Kim, Richard Fletcher, Kevin L. Moore

**Affiliations:** ^1^ Department of Radiation Medicine and Applied Sciences University of California San Diego La Jolla CA USA

**Keywords:** linear accelerator, quality management, online tools, technical support

## Abstract

To track linear accelerator performance issues, an online event recording system was developed in‐house for use by therapists and physicists to log the details of technical problems arising on our institution's four linear accelerators. In use since October 2010, the system was designed so that all clinical physicists would receive email notification when an event was logged. Starting in October 2012, we initiated a pilot project in collaboration with our linear accelerator vendor to explore a new model of service and support, in which event notifications were also sent electronically directly to dedicated engineers at the vendor's technical help desk, who then initiated a response to technical issues. Previously, technical issues were reported by telephone to the vendor's call center, which then disseminated information and coordinated a response with the Technical Support help desk and local service engineers. The purpose of this work was to investigate the improvements to clinical operations resulting from this new service model. The new and old service models were quantitatively compared by reviewing event logs and the oncology information system database in the nine months prior to and after initiation of the project. Here, we focus on events that resulted in an inoperative linear accelerator (“down” machine). Machine downtime, vendor response time, treatment cancellations, and event resolution were evaluated and compared over two equivalent time periods. In 389 clinical days, there were 119 machine‐down events: 59 events before and 60 after introduction of the new model. In the new model, median time to service response decreased from 45 to 8 min, service engineer dispatch time decreased 44%, downtime per event decreased from 45 to 20 min, and treatment cancellations decreased 68%. The decreased vendor response time and reduced number of on‐site visits by a service engineer resulted in decreased downtime and decreased patient treatment cancellations.

PACS numbers: 87.56.bd, 87.55.Qr

## I. INTRODUCTION

At the heart of modern radiation oncology are increasingly complex medical linear accelerators and ancillary devices such as imaging, tracking, and immobilization systems.^1^, ^2^ With increased complexity comes the challenge of maintaining effective quality management in the face of device malfunction and breakdown. Accordingly, tools and strategies for quality management, risk mitigation, and failure mode analysis have been adapted to the needs of radiation oncology.^3^, ^4^ Various organizations have published guidelines for ensuring effective quality management through the monitoring and periodic measurement of key functional parameters against specified tolerances.^5^, ^6^, ^7^, ^8^ Much of this information can now be recorded, distributed, and stored electronically, and the role of information technology (IT) in managing the data and integrating it into the radiation oncology workflow is increasingly important.^9^, ^10^, ^11^ Although much work can and has been done in‐house by other groups to develop custom IT solutions for radiotherapy clinical management,^12^, ^13^, ^14^, ^15^, ^16^, ^17^ collaborations with equipment vendors are recognized as an essential component in radiation oncology quality management.^(18)^

One element of quality management in the radiation therapy clinic is the service and support model for linear accelerators. In a typical arrangement, radiation therapists would report technical problems with the linear accelerator (or “machine”) to clinical medical physicists who, in turn, would report machine breakdown events verbally by telephone to the vendor's customer support dispatch center. This telephone interaction would involve the user, either a therapist or physicist, providing the identification of the machine, location of the installation site, and a description of the problem. Based on the customer's location and installed equipment, the call center staff would pass on this information to engineers at their Technical Support help desk. Together with the user, the Technical Support staff would attempt to diagnose the problem, and then coordinate repair actions with the clinical staff and the local service engineer assigned to that customer by the vendor.

We believed that the efficiency of linear accelerator support and service could be improved by four key innovations: 1) decreasing the number of steps in the event reporting chain, 2) decreasing repetitions and redundancies in the transmission of information related to the event, 3) electronically recording events for quality improvement and evaluation of service delivery, and 4) receiving remote support from individuals knowledgeable of our systems and their service history. In a collaboration between our institution and our linear accelerator vendor (Varian Medical Systems, Palo Alto, CA), these innovations have been incorporated into a new model of linear accelerator service and technical support. In the newly developed service model, all technical problems and nonclinical events related to machine operation are reported electronically directly to the vendor's Technical Support staff, who can then contact the user promptly to provide technical assistance and/or coordinate a response by the local service engineer. The initial event report is readily available to all recipients, eliminating the need to verbally redescribe the problem at each step in the communication chain. Furthermore, communication of specific event‐related information, such as installation site, machine type, and the nature of the problem, is accomplished electronically, minimizing voice communications which can be error prone and time‐consuming. Finally, by moving from a serial to a parallel event reporting distribution, response efficiency can be improved and overall response time decreased. For clinical operations, the most notable events are acute “machine‐down” problems that disallow treatments. The purpose of this work is to compare the new model of service and support to the old model in terms of performance and efficiency gains in service and support delivery, and to demonstrate its ability to improve clinical operations by minimizing disruptions due to therapy equipment problems.

## II. MATERIALS AND METHODS

### A. The Radiation Oncology Quality Reporting System

**Figure 1:**
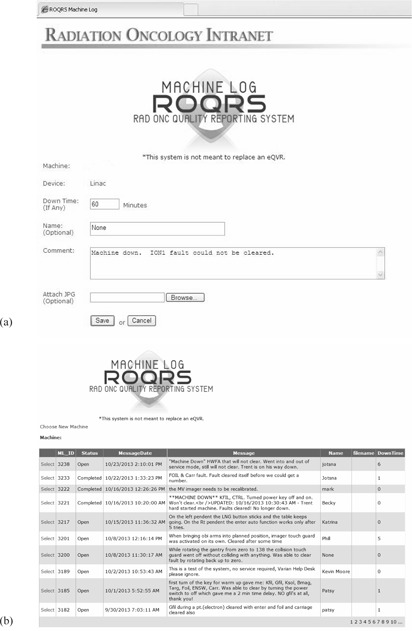
The ROQRS:ML user interface showing (a) user dialog for logging machine events that includes the machine identification, estimated downtime (if any), user name, free text narrative for describing the problem, and an optional image attachment that can be used for to include any on‐screen error messages; and (b) database view of ongoing error logs for a specific machine. The database that stores these error logs can be queried for specific key words, non‐zero downtime, etc.

Our institution employs in‐house developed software to report and manage quality control issues in the clinic, called the Radiation Oncology Quality Reporting System (ROQRS). Part of this system is an online tool called the ROQRS Machine Log (ROQRS:ML) that is used to communicate machine events in real‐time to clinical staff and to maintain a record of past and on‐going machine issues and follow‐up actions. Upon the occurrence of a machine event, such as an interlock or error message, the user, who is either a radiation therapist or a medical physicist, can generate a ROQRS:ML report through a Web‐based interface. A screenshot of a typical ROQRS:ML report is shown in Fig 1(a).

The information contained in the report is then transmitted electronically to an email distribution list that includes clinic staff such as operations managers, medical physicists, and senior therapists. In addition to spawning communications, the reports are also stored in a database that can be queried and modified with follow‐up information such as event resolution, total downtime, screen captures of relevant error messages, and technical support ticket numbers received by the hardware vendor (see Fig. 1(b)).

The ROQRS:ML event reporting system and database was implemented at our institution in October 2010 and was originally intended for use as a means of internal communication and recording of therapy equipment‐related events for quality tracking purposes. Starting in October 2012, a pilot project was initiated between our institution and our linear accelerator vendor to determine if the speed of technical support and service delivery could be improved if the ROQRS:ML event reports were sent by email to engineers at the vendor's Technical Support help desk, in addition to being distributed among key staff at our institution upon the occurrence of an event. A small group of the vendor's support engineers were specially assigned to receive the event notifications, selected from those that had familiarity with our institution's equipment, installation particulars, and past history of events.

Upon receipt of a ROQRS:ML event report, the vendor's support engineer would call the treatment machine directly to work with the attendant physicist. In some instances, vendor support engineers would call in even before the physicist arrived at the machine, beginning the troubleshooting process with radiation therapists until such time as the attendant physicist could get on the line. Each report and subsequent follow‐on response was distributed to all clinical physicists so that they remained within the email communication chain. At our institution, it is the responsibility of the scheduled clinical physicist of the day to attend to any machine problems in person at the unit if necessary, and to follow up on any problems and actions taken, particularly if they are based on otherwise undocumented verbal communications by phone. Furthermore, the ROQRS:ML database has functionality to indicate if problems are outstanding or resolved. A monthly incidents review meeting also reviews ROQRS:ML logs to ensure issues have been properly documented and to update an event's status as ongoing or closed.

### B. Evaluated parameters

The ROQRS:ML database of events between January 2012 and July 2013 was reviewed to quantify any changes in technical support and service delivery as a result of implementing the pilot project. This time period provides approximately equal evaluation periods before and after introduction of the new service model in October of 2012. For this analysis, we restricted our evaluation only to those ROQRS:ML events that involved a machine breakdown or “machine‐down”.

A machine‐down event was defined as a technical problem with the linear accelerator that interrupted, prevented, or required rescheduling or cancellation of patient treatments. For each machine‐down event, we recorded the time elapsed until receipt of a response from Technical Support staff. This included the time spent contacting the Technical Support help desk through the communication channels in use for each support model. If technical support was unavailable due to heavy call center demand, the call would be passed to the local service engineer. In these instances, time to a response was defined as the time to receipt of initial communication from the local service engineer. We also determined how the event was resolved, such as by a local service engineer visit, by physicists operating alone, or by liaising with the Technical Support staff. Finally, we determined the total clinical downtime.

The clinical downtime reported in ROQRS:ML events was initially estimated by clinical staff at the time of the event's resolution; to eliminate any human error in the reporting of this number, all reported downtime was later cross‐referenced against the time recorded in the ARIA Oncology Information System's database. The downtime was calculated from the time of the initial ROQRS:ML report to the first treatment‐related beam‐on time recorded in ARIA after resolution of the event. The total downtime before and after introduction of the new service model, the median weekly downtime, and the median downtime per event were then determined.

Technical support response time was defined as the time elapsed from the time of the event's electronic report to the time of initial contact by either Varian Technical Support or the local service engineer.

Treatment cancellations were defined as instances where patients were sent home without treatment because of an inoperable machine. The number of treatment cancellations was established by reviewing the patient treatment schedule within the ARIA database at the time of the event and counting the number of treatment cancellations. Cancellations did not include those patients who could be transferred to another machine in our institution for treatment. To determine the number of patients transferred to other machines, we used the ARIA database to review the dose record of patients treated during and after a machine event to determine which patients were treated on a machine other than that for which their treatment had originally been planned.

### C. Linear accelerators included in the pilot project

The ROQRS:ML system was used to report events occurring on any of four linear accelerators at our institution. All were manufactured by Varian Medical Systems and include a TrueBeam, Trilogy, 23iX RapidArc, and 21EX Clinac. The installation dates, energies, and installed features are listed in Table 1

Over the evaluated time period, the median number of patients on treatment in our clinic was 124, giving a typical workload on each machine of approximately 30 patients per day, operating from 6:30 a.m. to 6:00 p.m., five days per week. The “Key Features” column in Table 1 indicates the types of treatments that can be performed on each machine for the purposes of estimating the typical workload and complexity of daily treatments only; these features do not indicate their sole function within the clinic.

**Table 1:** Linear accelerators included in the electronic event reporting pilot project

*Machine Model*	*Installation Date*	*Approximate Age*	*Available Energies*	*Key Features*
TrueBeam	October, 2010	2 yr, 9 mo.	6X, 15X, 6FFF	VMAT, SRS, Gating
Trilogy 23iX RapidArc	May, 2005 June, 2009	8 yr, 2 mo. 4 yr, 1 mo.	6X, 15X, 6e, 9e, 12e, 16e, 20e 6X, 15X, 6e, 9e, 12e, 16e, 20e	SRS, Gating VMAT, SRS, Gating
21EX	May, 2005	8 yr, 2 mo.	6X, 15X, 6e, 9e, 12e, 16e, 20e	TBI, TSE

VMAT=volumetric modulated arc therapy; SRS=stereotactic radiosurgery; TBI=total body irradiation; TSE=total skin electron irradiation.

## III. RESULTS

Over 389 clinical days, there were 119 machine‐down events across the four linear accelerators, with 59 occurring before introduction of the new service model and 60 occurring after. Under the new model of service and support delivery, the median time to the initiation of a technical support response from the vendor decreased by 82%, from 45 to ~8min. The number of times that a local service engineer had to be emergently dispatched to carry out an on‐site repair was decreased by 44%, from 56 instances to 20 instances. The number of events that could be resolved by clinic staff, both with and without remote assistance from technical support, increased by 74%. Total downtime decreased by 47% from 3 h/week to 1.7 h/week. The median downtime per event decreased from 45 min to 20 min, a change of ‐56%. The number of treatment cancellations and/or patients who required rescheduling was decreased by 68%. The number of patients transferred to other treatment machines was 62, out of 348 patients affected by a machine down event, or 18%. The number of patients transferred was seen to increase from 27 to 35 after introduction of the new service model. A summary of all evaluated parameters is given in Table 2

**Table 2:** Performance data before and after introduction of the new support and service model

	*# of Events*	*Pre‐model*	*Post‐model*	*% Change*
Total Number of Clinic Days	389	203	186	
Total Number of Machine‐Down Events	119	59	60	
Median Time to Vendor Response (minutes)		45	8	‐82%
Resolved by On‐site Vendor Engineer	56	36	20	‐44%
Resolved by Clinic Staff	63	23	40	+74%
Resolved by Clinic Staff with Remote Assistance		2	22	
Total Downtime (minutes)		7391	3906	‐47%
Median Weekly Downtime (minutes)		60	20	‐67%
Median Downtime per Event (minutes)		45	20	‐56%
Treatment Cancellations	286	217	69	‐68%
Patients Transferred to Other Machines	62	27	35	+30%

## IV. DISCUSSION

The introduction of a real‐time event reporting system to our service and technical support model resulted in significantly reduced machine downtime, reduced patient treatment cancellations, and reduced visits by local service engineers.

The key factor in decreasing machine downtime was the reduced time to a response from the vendor's Technical Support staff. By simplifying the communication chain for the reporting of events, a machine‐down event could be resolved much faster. In the old model, much of the clinical downtime was spent in the vendor's initial contact process for receiving technical support. This entailed telephone calls through multiple points of contact and repeated verbal descriptions of the problem before receiving technical assistance related to the problem from support staff or a local service engineer.

Another major gain was the reduction in the number of times that service engineers had to be dispatched to resolve the technical issue. We attribute this to two factors. First, the improved communication link to Technical Support meant that support staff were aware of the event in real‐time. Greater confidence by clinical users in how to resolve technical events with remote assistance from the support staff meant that issues could often be resolved by clinical staff within minutes of their occurrence, both with and without remote assistance by Technical Support. A second factor contributing to improved response times was the assignment of dedicated engineers at the Technical Support help desk to this project. Because the same remote service personnel were interacting with local clinical staff on a daily basis, they could develop a familiarity with the institution's equipment, individual machine histories, and patterns of events.

It is a possibility that the initial selection of engineers who had prior familiarity with our institution and equipment configuration contributed to the improvement in service delivery. However, these were the same engineers providing call support to our institution prior to introduction of the reporting system. Thus, we maintain that the primary gains in delivery support response would have been due to the more efficient communications system.

The improved time to vendor support response had a follow‐on benefit in that clinical staff could arrive at a more accurate estimate of the duration of a machine‐down event and its impact upon the patient treatment schedule. This likely resulted in less patient cancellations because clinical staff knew with greater certainty when a machine was likely to be back in operation.

While a down machine is incredibly disruptive in any context, centers such as ours with multiple redundant linear accelerators can transfer patients off of inoperable machines, and this was often the case when a treatment unit was faced with significant downtime. From Table 2, approximately 18% of patients impacted by a machine‐down event could be transferred to another machine. The potential for reductions in patient cancellations at clinics operating just a single linear accelerator is thus underestimated in an analysis of treatment cancellations only. It should also be noted that at our center, the number of patients transferred was limited by a busy clinic schedule with little spare treatment capacity on other machines, as well as technique and equipment‐specific limitations to the easy transfer of patients to other machines.

This preliminary investigation only studied the effect of the new service model on machine‐down events and their impact on the clinic. For future work, we intend to quantify the anecdotal consensus among clinical staff that the remote support engineers' awareness of non‐urgent but persistent problems corresponded to more preventative maintenance. Addressing repetitive nonurgent events appeared to result in more preventative maintenance after clinic hours, though it remains to be demonstrated whether this resulted in a statistically significant reduction in the total number of events per machine. With a larger sample and a review of local service engineer maintenance reports, this analysis should allow a determination of what events most often led to machine downtime and if the repair time decreased for the same time of event. Ultimately this could quantify the benefits of this model to both clinic and vendor staff. Planned after‐hours maintenance is far less expensive to the clinic than unscheduled maintenance and is potentially less expensive to the vendor, as well.

## V. CONCLUSIONS

A new model of linear accelerator support and service delivery was implemented that employs real‐time electronic communication of machine events to dedicated staff at the vendor's Technical Support desk instead of a reliance on telephone and verbal communication. The old and new models were compared over equivalent clinical time periods and it was found that the new model lead to a decrease in vendor response time and a reduction in the number of on‐site visits required by service engineers. These performance gains resulted in decreased machine downtime and decreased patient treatment cancellations. This could prove to be an efficient and cost‐effective means for the delivery of linear accelerator support, should vendors expand their service offerings to include this form of customer care.

## ACKNOWLEDGMENTS

This work was done in collaboration Varian Medical Systems. The pilot project was developed within the existing linear accelerator service agreement between the vendor and our institution, and no extra financial compensations were provided.
